# [Neratinib + Valproate] exposure permanently reduces ERBB1 and RAS expression in 4T1 mammary tumors and enhances M1 macrophage infiltration

**DOI:** 10.18632/oncotarget.23681

**Published:** 2017-12-26

**Authors:** Laurence Booth, Jane L. Roberts, Rumeesa Rais, John Kirkwood, Francesca Avogadri-Connors, Richard E. Cutler, Alshad S. Lalani, Andrew Poklepovic, Paul Dent

**Affiliations:** ^1^ Department of Biochemistry and Molecular Biology, Virginia Commonwealth University, Richmond, VA 23298, USA; ^2^ Department of Medicine, Virginia Commonwealth University, Richmond, VA 23298, USA; ^3^ Puma Biotechnology Inc., Los Angeles, CA 90024, USA; ^4^ University of Pittsburgh Cancer Institute Melanoma and Skin Cancer Program, Hillman Cancer Research Pavilion Laboratory L1.32c, Pittsburgh, PA 15232, USA

**Keywords:** autophagy, receptor tyrosine kinase, neratinib, valproate

## Abstract

The irreversible ERBB1/2/4 inhibitor neratinib has been shown *in vitro* to rapidly reduce the expression of ERBB1/2/4 and RAS proteins via autophagic/lysosomal degradation. We have recently demonstrated that neratinib and valproate interact to suppress the growth of 4T1 mammary tumors but had not defined whether the [neratinib + valproate] drug combination, in a mouse, had altered the biology of the 4T1 cells. Exposure of 4T1 mammary tumors to [neratinib + valproate] for three days resulted, two weeks later, in tumors that expressed less ERBB1, K-RAS, N-RAS, indoleamine-pyrrole 2,3-dioxygenase (IDO-1), ornithine decarboxylase (ODC) and had increased Class I MHCA expression. Tumors previously exposed to [neratinib + valproate] grew more slowly than those exposed to vehicle control and contained more CD8+ cells and activated NK cells. M1 but not M2 macrophage infiltration was significantly enhanced by the drug combination. *In vitro* exposure of 4T1 tumor cells to [neratinib + valproate] variably reduced the expression of histone deacetylases 1-11. *In vivo*, prior exposure of tumors to [neratinib + valproate] permanently reduced the expression of HDACs 1-3, 6 and 10. Combined knock down of HDACs 1/2/3 or of 3/10 rapidly reduced the expression IDO-1, and ODC and increased the expression of MHCA. H&E staining of normal tissues at animal nadir revealed no obvious cyto-architectural differences between control and drug-treated animals. We conclude that [neratinib + valproate] evolves 4T1 tumors to grow more slowly and to be more sensitive to checkpoint immunotherapy antibodies.

## INTRODUCTION

The recently FDA approved ERBB1/2/4 inhibitor neratinib at physiologic concentrations was shown by our group to rapidly down-regulate the expression of ERBB1/2/3/4, c-MET, PDGFRα and mutant K-/N-RAS proteins [1, 2]. When we microscopically examined neratinib-treated cells at 60× magnification we observed the appearance of vesicles inside the cell, close to the plasma membrane, that contained ERBB family receptors and mutant RAS proteins. These vesicles co-stained for Beclin1 and/or cathepsin B and LAMP2. i.e. neratinib was utilizing autophagosomes and autolysosomes to cause the degradation of growth factor receptors and RAS proteins. Other investigators have made similar observations for neratinib, specifically examining the down-regulation of ERBB2 expression [3]. The precise mechanisms by which neratinib rapidly causes the internalization of receptor tyrosine kinases and then their subsequent degradation are unknown.

We determined that neratinib and the HDAC inhibitor sodium valproate interacted to suppress the growth of 4T1 mammary tumors growing in their syngeneic BALB/c host mouse and that this drug combination could also enhance the efficacy of an anti-PD1 antibody against the 4T1 tumors [1]. From *in vitro* studies, we determined that the ability of neratinib and valproate to interact to kill and to alter tumor cell immunogenicity were in part dependent on the drug combination inducing autophagosome formation [1, 2]. This finding is like those we recently made using the drug combinations of [pemetrexed + sildenafil] and [pazopanib + HDAC inhibitors] that also require autophagosome formation to both kill and to enhance tumor cell immunogenicity [4–6]. The autophagy-dependent alteration in tumor cell immunogenicity biomarkers *in vitro* was directly associated to the ability of autophagy to also degrade and reduce the expression of HDAC proteins, which impacted on transcription, e.g. HDACs 1 and 3, as well as on protein stability/activity, e.g. HDAC6 [1, 2, 4–6]. Thus [neratinib + valproate], through promoting the degradation of HDACs1, 3 and 10, was shown to enhance the expression of Class I MHCA whilst simultaneously through the same mechanism reducing the expression of PD-L1 and ornithine decarboxylase. However, we have as yet not demonstrated that *in vivo* drug exposure, via HDAC regulation, can be linked to changes in immune system biomarker expression in drug-treated tumor cells.

The present studies were performed to further define the impact of [neratinib + valproate] exposure on the expression of immunological biomarkers, HDAC and other survival regulatory proteins and the infiltration of immune cells into treated tumors. We discovered that prior [neratinib + valproate] exposure results in a re-programing of tumor cells that survived and re-grew, with cells expressing less ERBB1, K-RAS and N-RAS, and having reduced the expression of multiple HDAC proteins that regulate tumor cell immunogenicity.

## RESULTS

**Figure 1:**
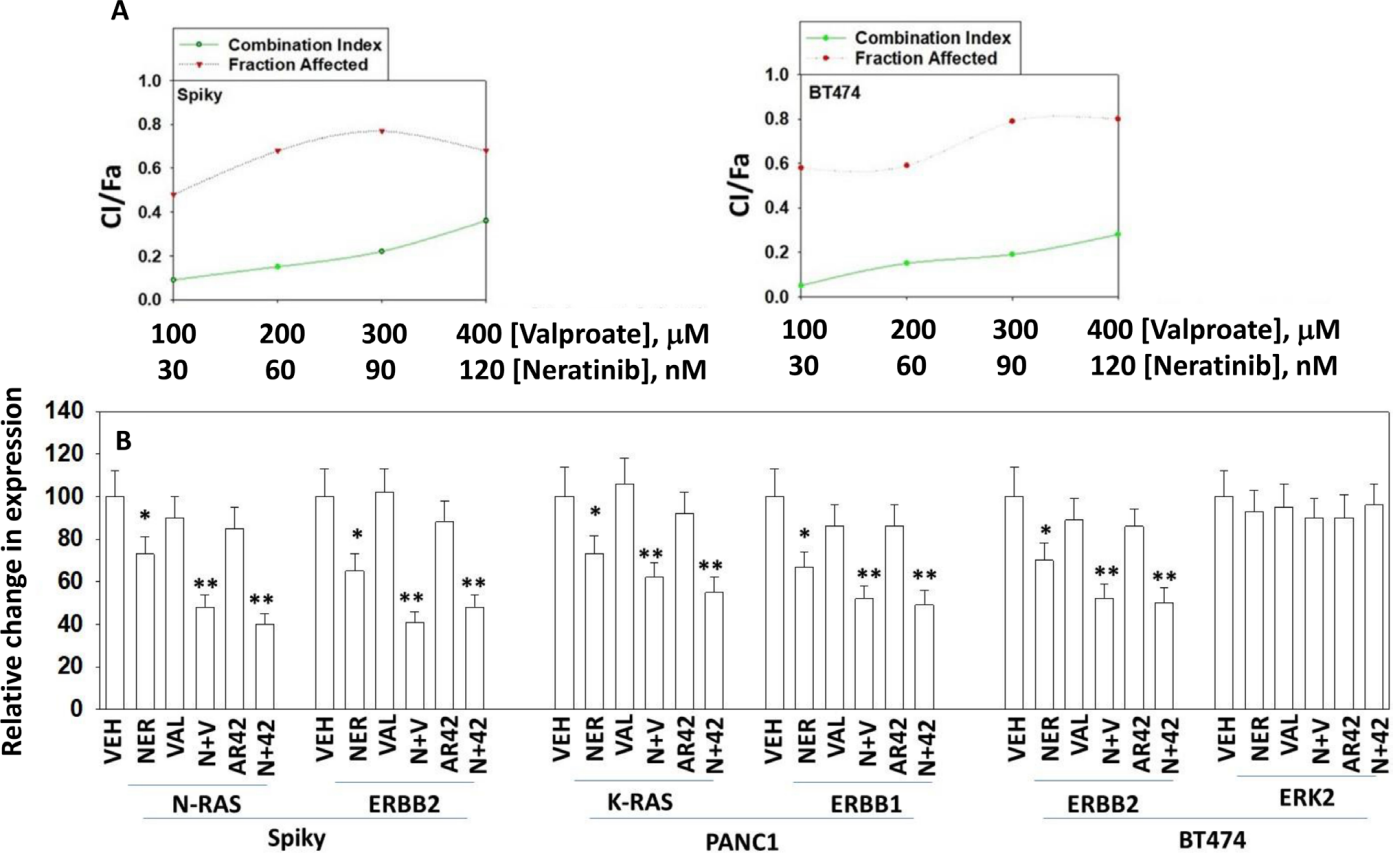
Neratinib and HDAC inhibitors synergize to kill cancer cells (**A**) Spiky cells and BT474 cells were plated in 6-well plates in sextuplicate as individual cells (500 cells per well). After 12 h the cells were treated with vehicle control, neratinib, niraparib or the drugs combined, at the indicated concentrations in the figure, at a fixed ratio. After 24 h, the media is removed, the cells washed with warm drug-free media, and fresh drug-free media placed on the cells. After 10 days, colonies of > 50 cells have formed and the cells are fixed in place and stained with crystal violet. The plating efficiency under each treatment condition is determined and the fraction affected determined. Synergy was determined using the Calcusyn for Windows program using the method of Cho and Talalay (*n* = 2 independent studies in sextuplicate). A combination index of less than 1.00 indicates a synergy of drug interaction. The colony formation/fraction affected are plotted on the same graph. (**B**) Spiky, PANC1 and BT474 cells were treated with vehicle control, neratinib (50 nM), sodium valproate (250 µM), AR42 (600 nM) or the drugs in combination for 6 h as indicated. The cells were fixed in place and immunostaining performed to determine the expression of the indicated proteins at 10× magnification (data from multiple separate images & treatments ±SEM) ^*^p < 0.05 less than vehicle control; ^**^p < 0.05 less than neratinib alone value.

**Figure 2:**
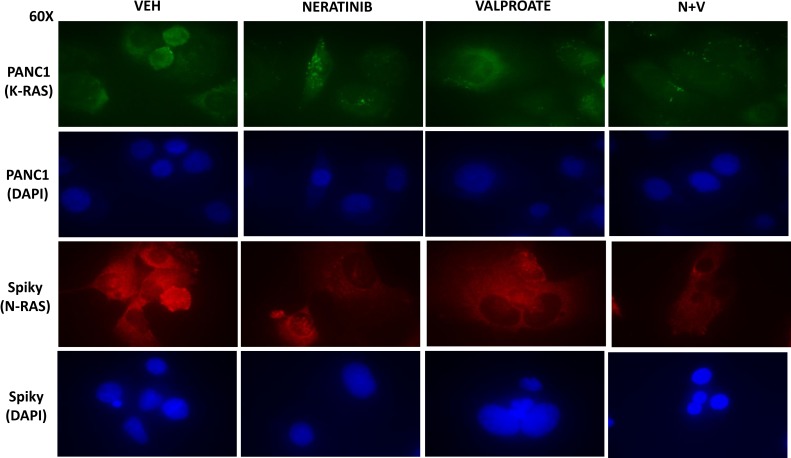
Neratinib and HDAC inhibitors interact to reduce the expression of mutant N-RAS and mutant K-RAS Spiky and PANC1 cells that express a mutant N-RAS and a mutant K-RAS, respectively, were treated with vehicle control, neratinib (50 nM), sodium valproate (250 µM), or the drugs in combination for 6h as indicated. The cells were fixed in place and immunostaining performed with DAPI counter-stain to determine the expression levels and cellular localization of N-RAS and K-RAS at 60× magnification.

In median dose effect isobologram colony formation assays where cells were transiently exposed to drugs for 24 h and then permitted to form colonies in the absence of drugs, neratinib and sodium valproate interacted in a synergistic fashion to kill ovarian (Spiky) and mammary (BT474) tumor cells, with combination index values of less than 1.0 (Figure 1A). We initially performed descriptive *in vitro* studies to define changes in ERBB1/2, K-RAS and N-RAS expression caused by the drugs as single agents and in combination after 6 hours of exposure. Neratinib acted to significantly reduce the expression of ERBB1, ERBB2, K-RAS and N-RAS, but not that of ERK2 (Figure 1B). In Spiky, PANC1 and BT474 cells neither sodium valproate nor AR42 as single agents were as capable of reducing the expression of ERBB1, ERBB2, K-RAS and N-RAS when compared to neratinib. This is different from our prior studies in afatinib-resistant H1975 cells [7]. Both valproate and AR42 significantly enhanced the ability of neratinib to reduce the expression of ERBB1, ERBB2, K-RAS and N-RAS. Our prior studies using PANC1 pancreatic cancer cells had demonstrated that neratinib caused ERBB1 and K-RAS to rapidly localize in vesicles within the cell that co-stained for phosphorylated ATG13 and also with cathepsin B. At 60× magnification we discovered that neratinib as a single agent reduced overall K-RAS and N-RAS expression and caused the staining of both RAS proteins to become punctate (Figure 2). Valproate did not appreciably alter the levels of K-RAS/N-RAS but enhanced the ability of neratinib to down-regulate expression of the RAS proteins.

**Figure 3:**
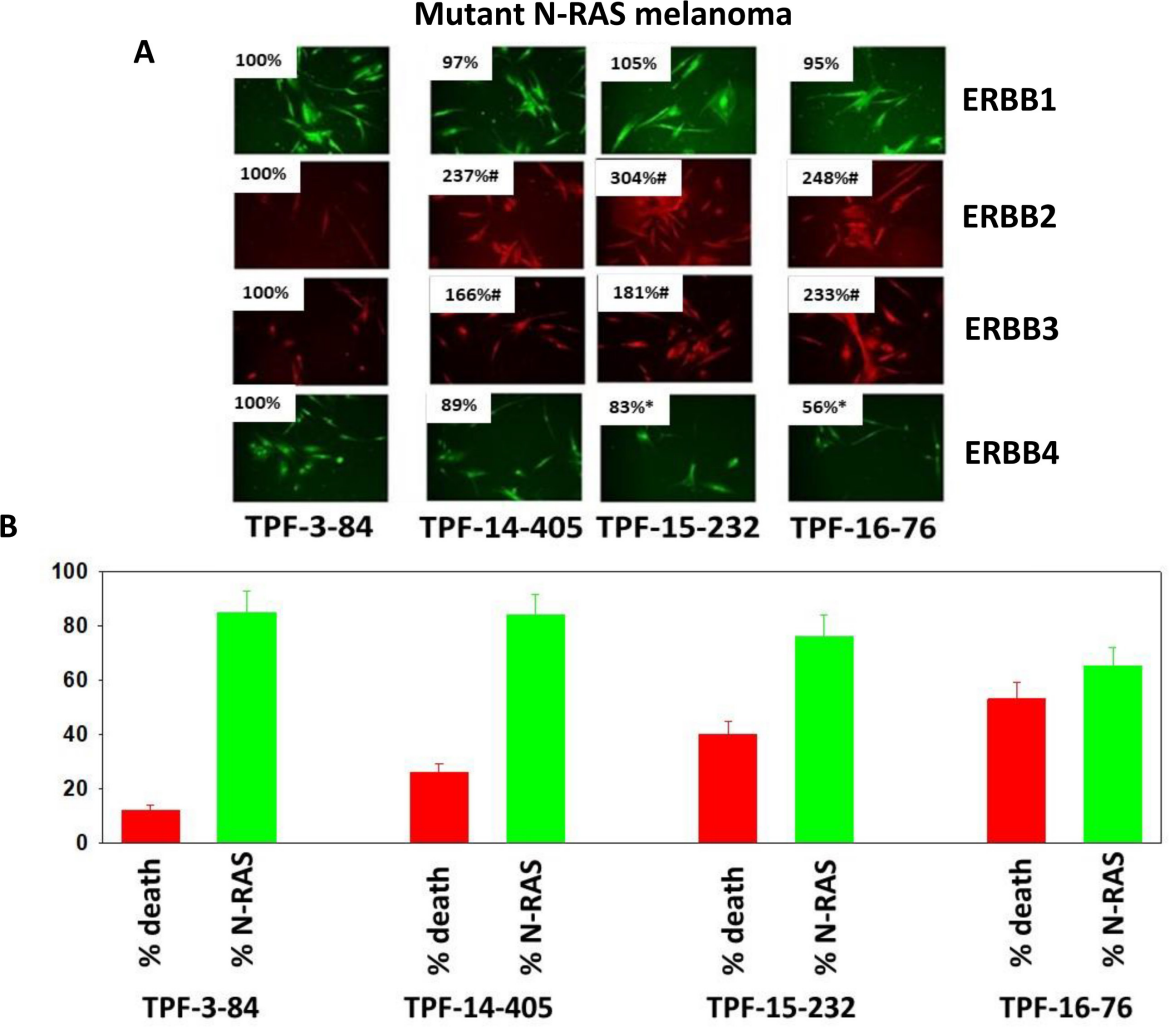
Neratinib and valproate reduce N-RAS expression in PDX melanoma isolates and variably enhance tumor cell killing (**A**) Mutant N-RAS expressing PDX isolates of melanoma from the University of Pittsburgh cell bank were grown for 24 h, fixed, and stained to determine the protein expression of ERBB1, ERBB2, ERBB3 and ERBB4. (*n* = 3 replicates of 40 cells each assessed for their fluorescence intensity ± SEM). ^*^p < 0.05 less than value in TPF-3-84; ^#^*p* < 0.05 greater than value in TPF-3-84. (**B**) RED: Melanoma isolates were treated with vehicle control or with [neratinib (50 nM) + valproate (250 µM)] for 24 h. Cell viability after 24 h exposure was determined by live/dead assay. (*n* = 3 ± SEM) ^#^*p* < 0.05 greater than vehicle control. GREEN: Melanoma isolates were treated with vehicle control or with [neratinib (50 nM) + valproate (250 µM)] for 6h. Cells were fixed in place and immunostaining performed to determine the expression level of N-RAS. (*n* = 3 replicates of 40 cells each assessed for their fluorescence intensity ± SEM). ^*^p < 0.05 less than value in vehicle control.

One question we asked ourselves was whether we could observe a simple correlation between neratinib protein targets that are down-regulated, e.g. ERBB2 and N-RAS, and the ability of neratinib in combination with valproate to kill the tumor cells. The protein expression of ERBB1 and ERBB4 in the TPF-3-84 melanoma isolate was similar to the expression levels found in the TPF-14-405, TPF-15-232 and TPF-16-76 isolates (Figure 3A). However, the protein levels of ERBB2 and ERBB3 were significantly lower in TPF-3-84 compared to the other isolates tested. Although the levels of mutant N-RAS expression in all of the melanoma isolates were not significantly different, the ability of [neratinib + valproate] to reduce N-RAS expression varied with TPF-3-84 and TPF-14-405 presenting with a significantly lower N-RAS down-regulation effect compared to the down regulation effect in TPF-15-232 and TPF-16-76 (Figure 3B, lower). In parallel, we determined the lethality of [neratinib + valproate] in each isolate: drug combination lethality was lowest in TPF-3-84; killing was significantly elevated in TPF-14-405 with the highest levels of killing observed in TPF-15-232 and TPF-16-76 (Figure 3B, upper). Thus TPF-3-84, with the lowest expression of ERBB2 and ERBB3, and with a weaker down-regulation of mutant N-RAS exhibited the lowest induction of cell killing by the drug combination. However, TPF-14-405, with a similar level of N-RAS down-regulation to TPF-3-84, but approximately twice as much basal ERBB2/ERBB3 expression, was more effectively killed by the drug combination, from 12% death in TPF-3-84 to 26% death in TPF-14-405. Studies beyond the scope of the present manuscript will be required to fully understand the correlation between receptor and RAS down-regulation and tumor cell killing caused by [neratinib + valproate].

**Figure 4:**
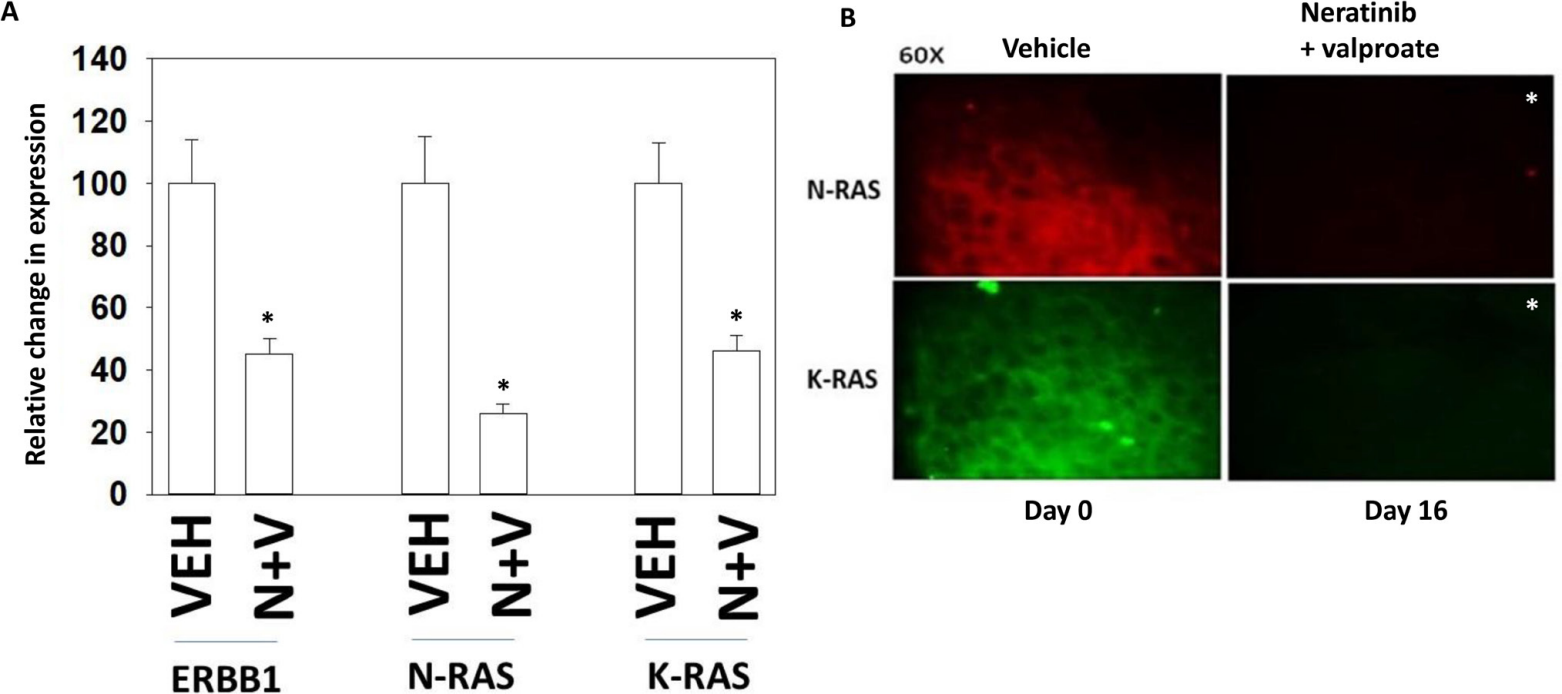
Tumors previously exposed to [neratinib + valproate] have a sustained reduction in the expression of ERBB1, N-RAS and K-RAS (**A**) 4T1 tumors, removed on Day 16 were fixed, paraffin embedded and sectioned (5 μm). Sections were renatured, blocked and immunostaining performed to determine the expression of ERBB1, N-RAS and of K-RAS. Images were taken at 10× magnification. (data from multiple separate images & treatments ± SEM) ^*^p < 0.05 less than vehicle control. (**B**) 4T1 tumors, removed on Day 16 were fixed, paraffin embedded and sectioned (5 μm). Sections were renatured, blocked and immunostaining performed to determine the expression of N-RAS and K-RAS. Images were taken at 60× magnification. (data from multiple separate images & treatments ± SEM) ^*^p < 0.05 less than vehicle control.

We have recently published that neratinib and sodium valproate interact *in vivo* to suppress the growth of 4T1 TNBC mouse mammary tumors [1]. This occurred without any apparent observable toxicity in normal tissues previously exposed to the drug combination as judged by H&E staining (Supplementary Figure 1). Animal body mass and animal behavior were not altered by exposure to the drug combination (data not shown). In tumors removed from the animal on Day 16, thirteen days after cessation of the three-day drug treatment, we observed in 5 μm immuno-stained tumor sections that the expression of K-RAS, N-RAS and ERBB1 were all significantly reduced (Figure 4A). We examined the same sections again at 60× magnification and noted that whereas under vehicle control conditions the staining for RAS proteins was localized around the periphery of the cell, in those tumors previously exposed to [neratinib + valproate] the much weaker staining appeared to be more evenly distributed all over the cell (Figure 4B).

**Figure 5:**
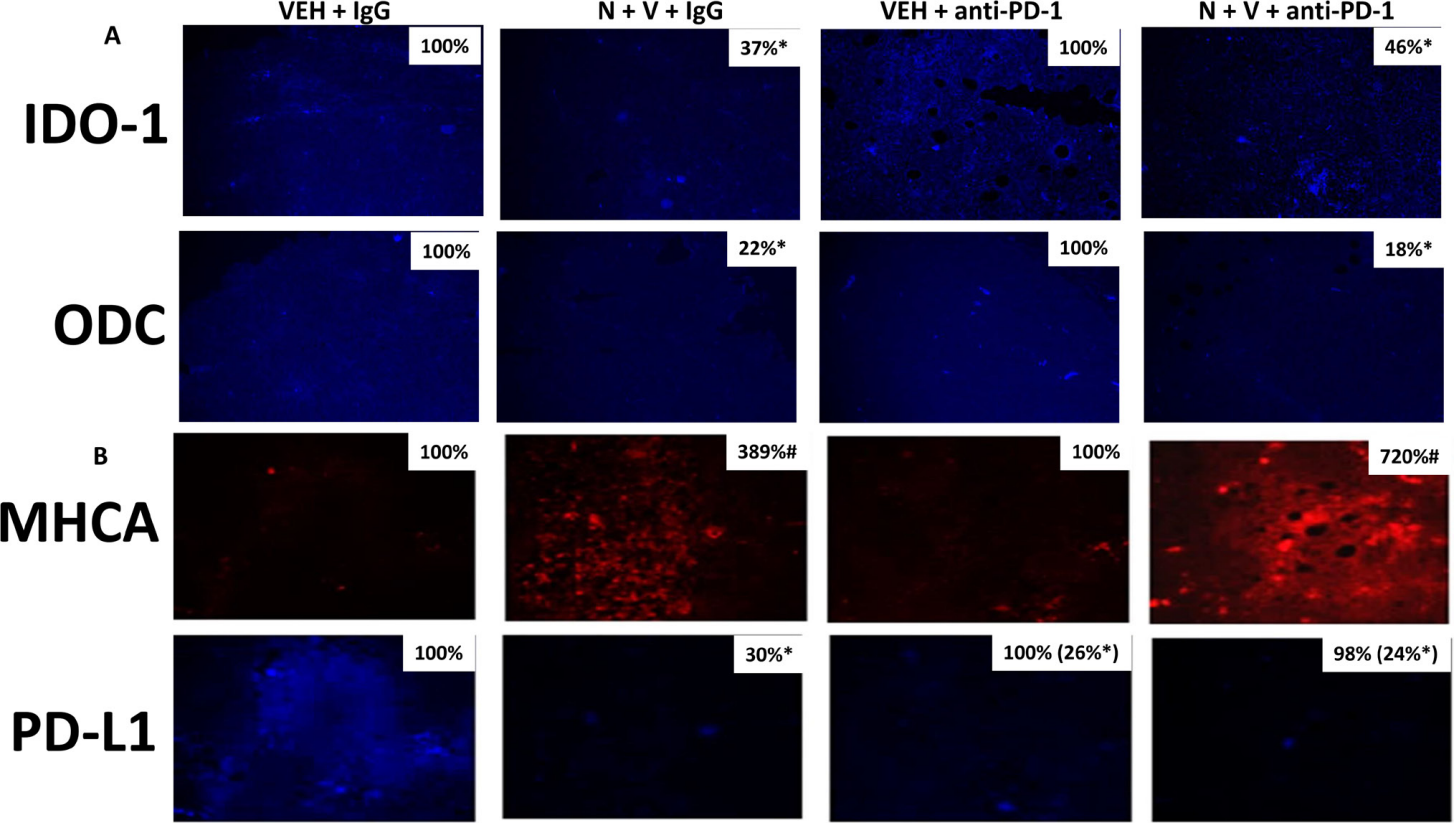
Prior [neratinib + valproate] exposure reduces IDO-1, ODC and PD-L1 expression and enhances MHCA expression in 4T1 tumors (**A**) 4T1 tumors, removed on Day 16 were fixed, paraffin embedded and sectioned (5 μm). Sections were renatured, blocked and immunostaining performed to determine the expression of IDO-1 and of ODC. Images were taken at 10× magnification. (data from multiple separate images & treatments ± SEM) ^*^p < 0.05 less than vehicle control. (**B**) 4T1 tumors, removed on Day 16 were fixed, paraffin embedded and sectioned (5 μm). Sections were renatured, blocked and immunostaining performed to determine the expression of MHCA and of PD-L1. Images were taken at 10× magnification. (data from multiple separate images & treatments ± SEM) ^*^p < 0.05 less than vehicle control.

We have previously discovered that exposure of 4T1 tumors to [neratinib + valproate] enhances the anti-tumor efficacy of a subsequent administration of an anti-PD1 checkpoint inhibitory antibody [1]. Prior exposure of 4T1 tumors with [neratinib + valproate] significantly reduced the tumor expression of IDO-1 and ODC which was not altered in tumors also exposed to the anti-PD1 antibody (Figure 5A). Prior exposure of 4T1 tumors to [neratinib + valproate] enhanced the expression of Class I MHCA and in parallel, reduced the expression of PD-L1; the reduced PD-L1 expression correlates to the enhanced anti-tumor efficacy of the anti-PD1 antibody administration (Figure 5B). Interestingly, administration of the anti-PD1 antibody profoundly reduced PD-L1 expression in the 4T1 tumors, and the combination of the drugs with the antibody further enhanced MHCA levels. These data argue that our drug/antibody exposures are altering the tumor cells and the tumor microenvironment to a state more conducive to checkpoint immunotherapy.

**Figure 6:**
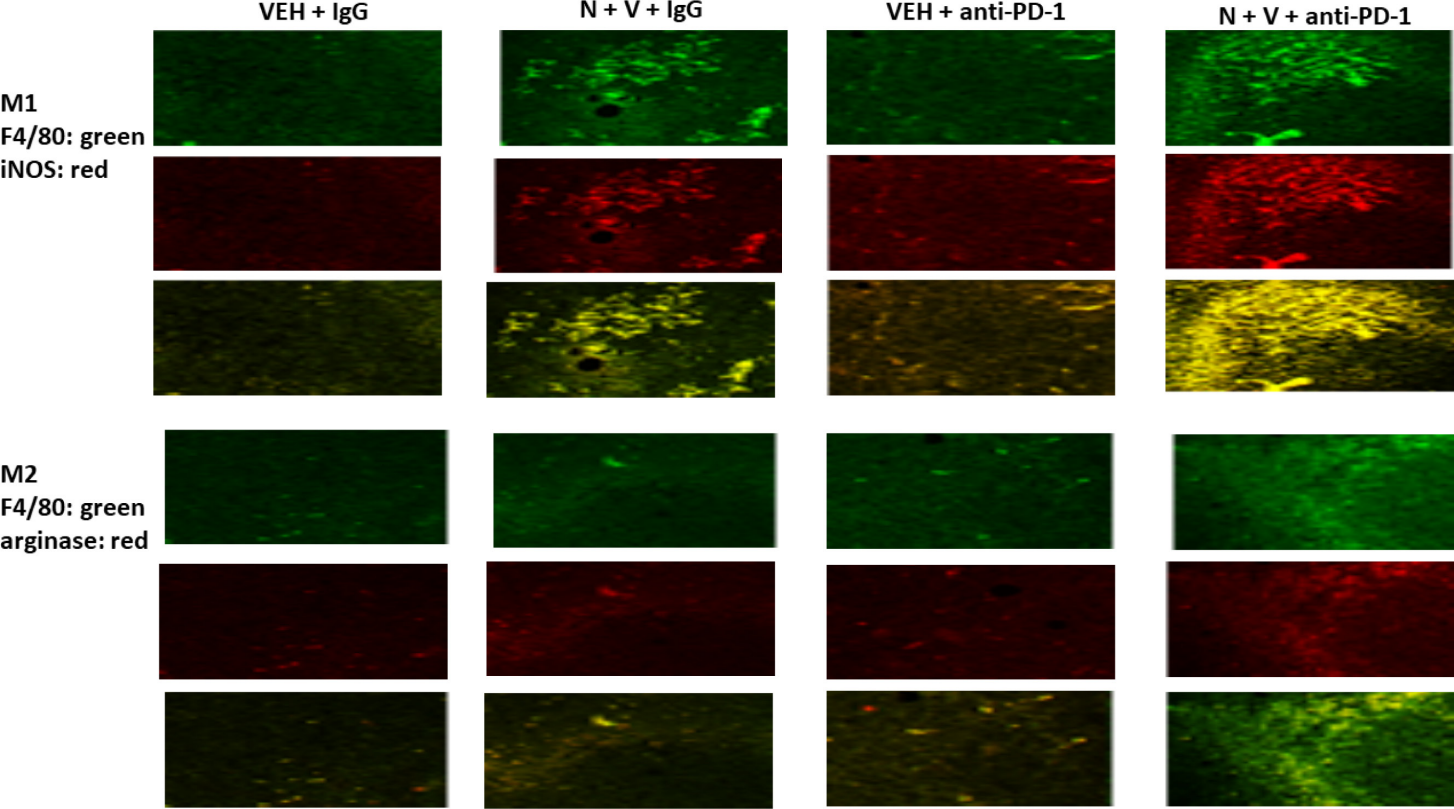
Prior [neratinib + valproate] exposure enhances the invasion of active NK cells, activated T cells and M1 polarized macrophages into 4T1 tumors 4T1 tumors, removed on Day 16 were fixed, paraffin embedded and sectioned (5 μm). Sections were renatured, blocked and immunostaining performed to determine the expression of F4/80, iNOS and arginase within the tumor. Images were taken at 10× magnification. Fluorescence intensity staining levels for each antibody in multiple sections were obtained to determine the relative staining intensities under each condition of F4/80, iNOS and arginase (*n* = 3 ± SEM, *p* < 0.05 staining intensity of iNOS greater than intensity of arginase).

**Figure 7:**
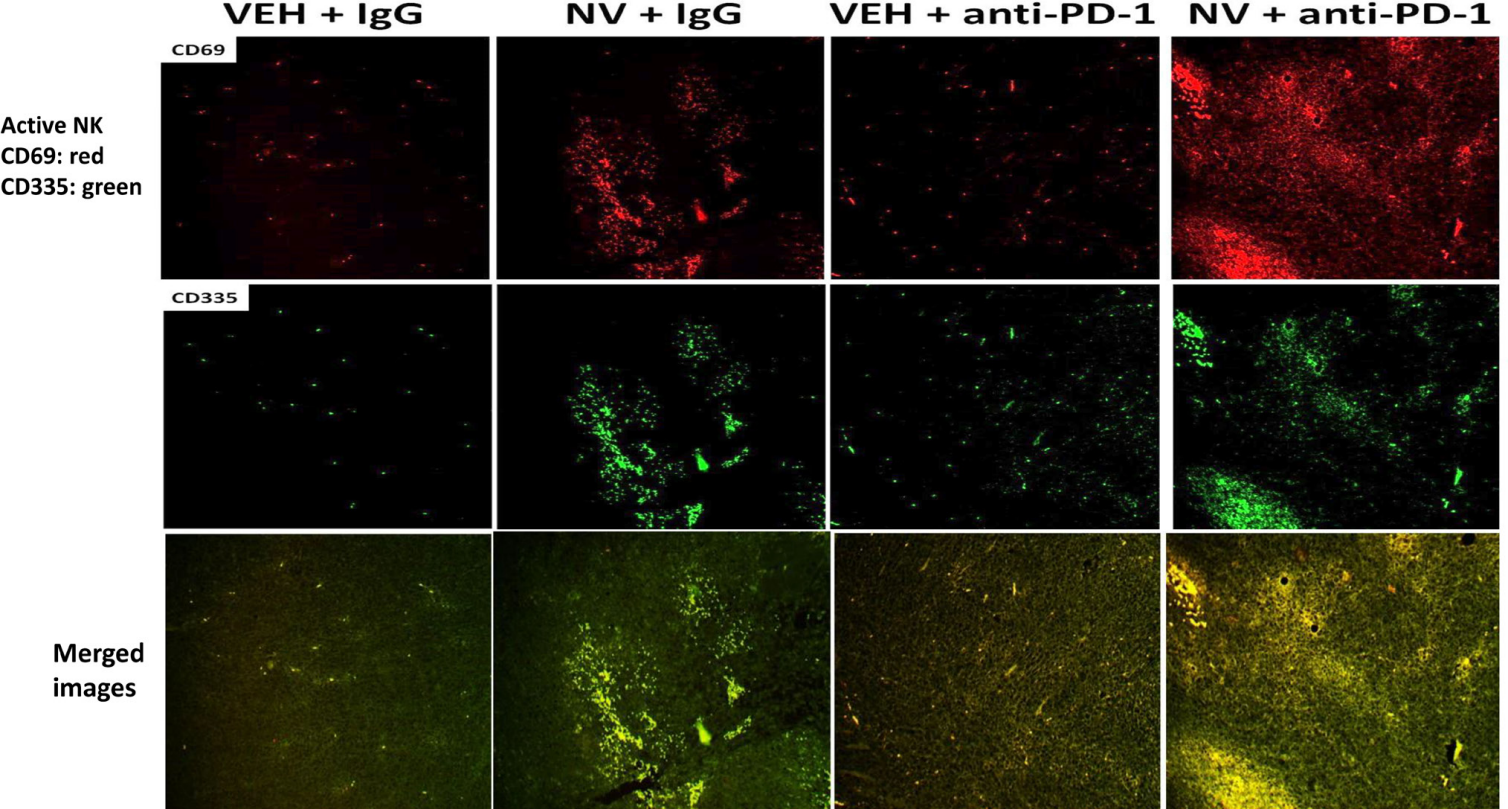
[Neratinib + valproate] promotes activated NK cell tumor infiltration, that is enhanced by an anti-PD-1 antibody 4T1 tumors, removed on Day 16 were fixed, paraffin embedded and sectioned (5 μm). Sections were renatured, blocked and immunostaining performed to determine the expression of CD69 and of CD335 within the tumor. Images were taken at 10× magnification and presented with elevated contrast to remove low-level background staining.

**Figure 8:**
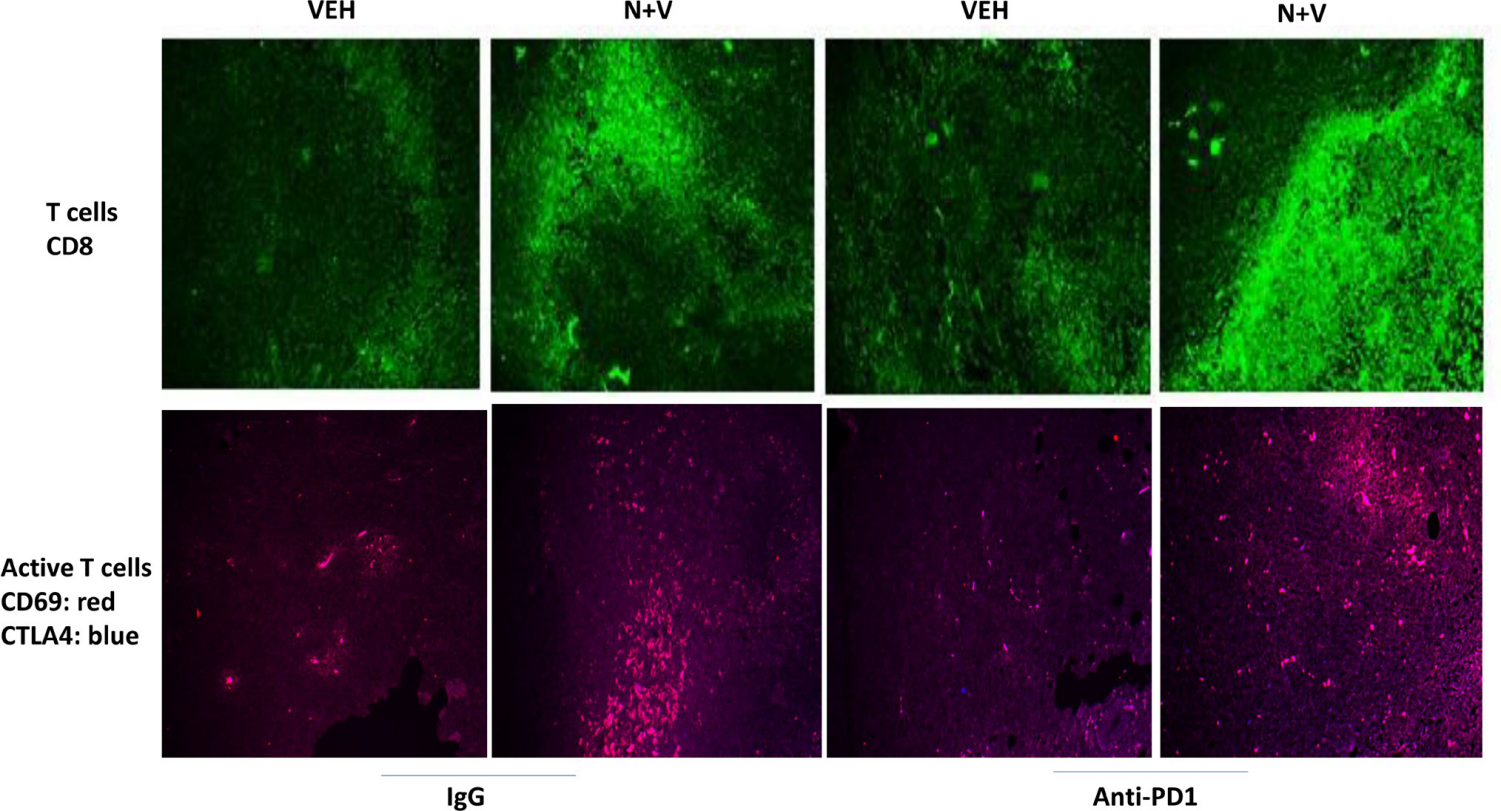
[Neratinib + valproate] increases the levels of CD8+ and CD69+CTLA4+ cells in 4T1 tumors 4T1 tumors, removed on Day 16 were fixed, paraffin embedded and sectioned (5 μm). Sections were renatured, blocked and immunostaining performed to determine the expression of CD8 or of [CD69 + CTLA4] within the tumor. Images were taken at 10× magnification. Using Adobe Photoshop 6.0 the staining/co-staining images for CD8+ and [CD69+CTLA4+] were obtained. Red + Green staining = yellow; Red + Blue staining = mauve.

We next determined whether our treatments had altered the infiltration/levels of immune cells into the tumors. Macrophage infiltration into a tumor can either facilitate anti-tumor effects, performed by M1 polarized macrophages, or can promote tumor growth, performed by M2 polarized macrophages [8]. Prior treatment of tumors with [neratinib + valproate] increased the number M1 polarized macrophages and M2 polarized macrophages in the tumor (Figure 6). For M1 macrophages, over 80% of the F4/80 green staining cells co-localized with iNOS whereas less than 20% of the F4/80 green staining cells co-localized with the M2 macrophage biomarker arginase (*p* < 0.05). Neratinib-valproate exposure resulted in enhanced infiltration of cells that stained strongly for CD69 and CD335 (activated natural killer cells) (Figure 7). The levels of CD69+ CD335+ cells within the tumors was enhanced when an anti-PD1 antibody was added to the drug combination. Prior exposure to the [neratinib + valproate] drug combination increased the numbers of CD8+ T cells and CD69+ CTLA4+ activated T cells in tumors (Figure 8). Thus, prior exposure of tumors to [neratinib + valproate] promotes anti-tumor immune responses through multiple overlapping mechanisms including infiltrating NK cells, M1 macrophages and reduced IDO-1 and ODC expression.

**Figure 9:**
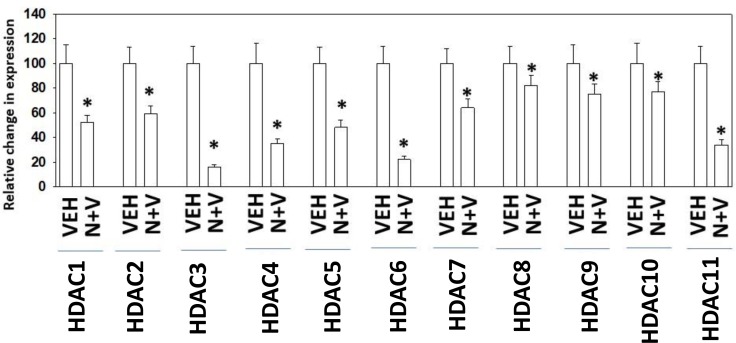
Exposure of 4T1 cells to [neratinib + valproate] rapidly reduces HDAC expression *in vitro* 4T1 cells were treated with vehicle control, neratinib (50 nM), sodium valproate (250 µM) or the drugs combined for 6 h. Cells were fixed in place and immunostaining performed to determine the expression levels of HDACs 1-11. (*n* = 3 replicates of 40 cells each assessed for their fluorescence intensity ± SEM). ^*^p < 0.05 less than value in vehicle control.

**Figure 10:**
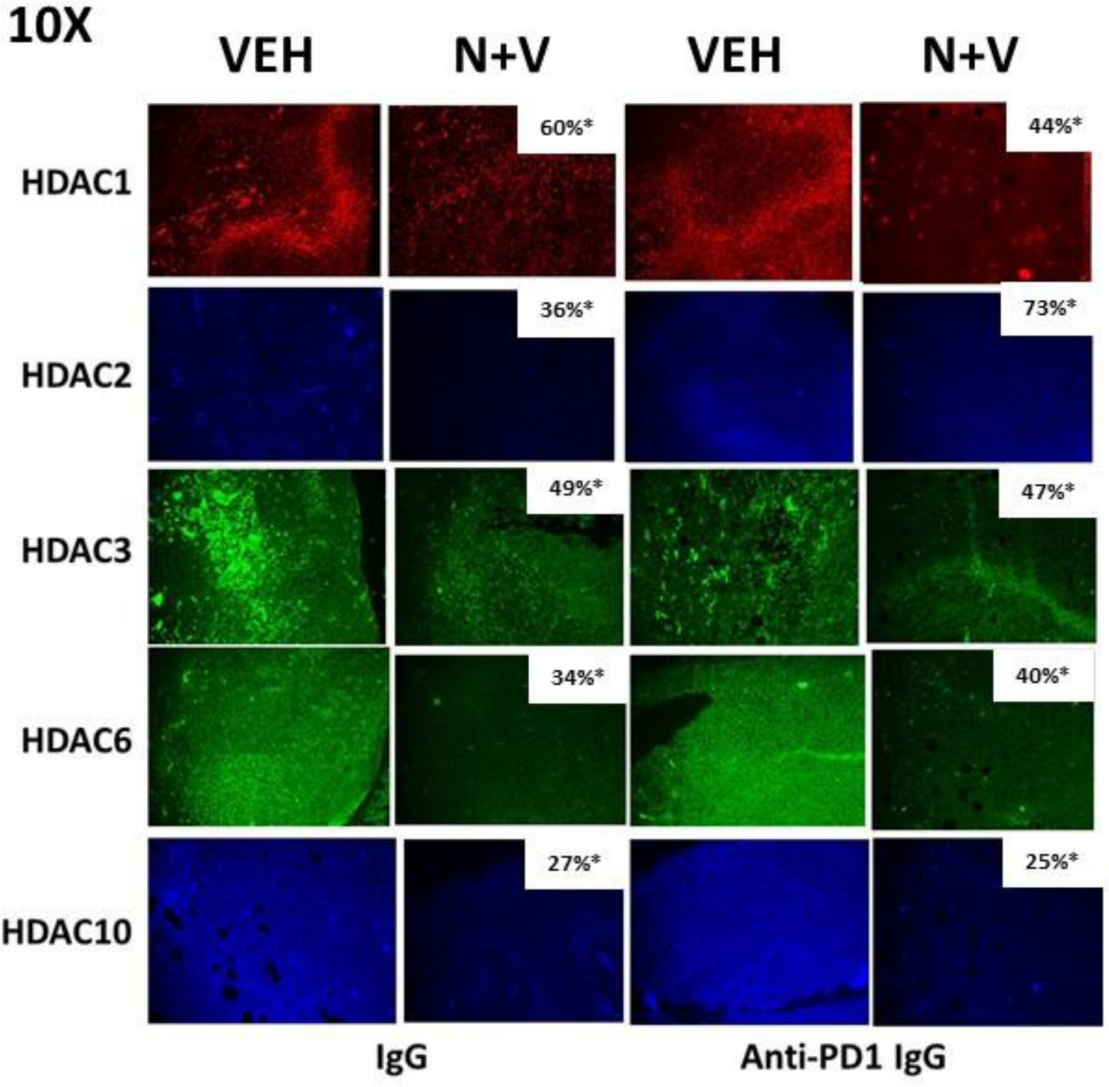
Prior exposure of 4T1 tumors *in vivo* to [neratinib + valproate] permanently reduces HDAC expression in the surviving 4T1 cells 4T1 tumors, removed on Day 16 were fixed, paraffin embedded and sectioned (5 μm). Sections were renatured, blocked and immunostaining performed to determine the expression of HDAC1, HDAC2, HDAC3, HDAC6 and HDAC10 within the tumor. Images were taken at 10× magnification and presented with elevated contrast to remove low-level background staining. Fluorescence intensity staining levels for each antibody in multiple sections were obtained to determine the relative staining intensities the HDACs (*n* = 3 ± SEM). ^*^p < 0.05 less than corresponding value in vehicle control tumors.

Prior *in vitro* studies from our laboratory have shown that drug combinations that induce autophagosome formation, e.g. [pemetrexed + sildenafil], [pazopanib + HDAC inhibitors], cause the proteolytic degradation of histone deacetylase proteins [4–6]. *In vitro* treatment of 4T1 rodent TNBC cells for 6h with [neratinib + valproate] variably reduced the expression of HDACs 1-11 (Figure 9). We then performed additional immuno-histochemical analyses to determine whether prior exposure of 4T1 tumors to [neratinib + valproate] also altered the expression of HDACs. Prior exposure to [neratinib + valproate] significantly reduced the expression of HDACs 1-3, 6 and 10 in tumor sections (Figure 10). Thus, not only can the drug combination reduce HDAC levels *in vitro* in the short term (6 h), but the combination can re-program surviving tumor cells *in vivo* to make less of these HDACs in tumors over a much longer period of time (weeks).

**Figure 11:**
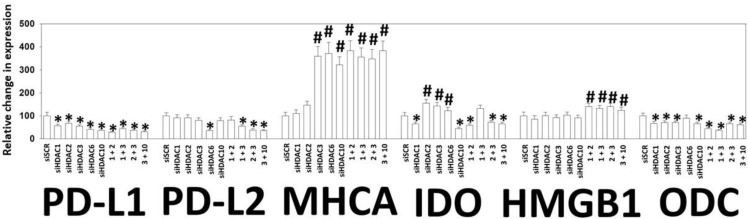
Knock down of HDACs reduces PD-L1, PD-L2, IDO-1 and ODC expression and enhances MHCA expression in 4T1 cells 4T1 cells were transfected with an siRNA control (siSCR) or with siRNA molecules to knock down the expression of HDAC1, HDAC2, HDAC3, HDAC6, HDAC10, or with combinations of HDAC knock down as indicated in the Figure. Twenty-four h after transfection cells were fixed in place and immunostaining performed to determine the staining intensity under each knock down condition of PD-L1, PD-L2, MHCA, IDO-1, HMGB1 and ODC. (data from multiple separate images & treatments ± SEM) ^*^p < 0.05 less than siSCR control; ^#^*p* < 0.05 greater than siSCR control.

Based on the data in Figures 5–9, we next attempted to molecularly associate changes in HDAC expression with changes in the expression of immuno-regulatory proteins. As presented in Figure 11, knock down of HDACs 1-3 and 10 reduced the expression of PD-L1, IDO-1 and ODC and increased the expression of MHCA (Figure 11). HDAC6 is a cytosolic HDAC that regulates heat shock protein 90 (HSP90) function, with inhibition of HDAC6 reducing the chaperoning function of HSP90 [9]. Knock down of HDAC6 reduced the expression of PD-L1, PD-L2, IDO-1 and enhanced the levels of MHCA. This finding with HDAC6 is different from our data in B16 melanoma cells and afatinib-resistant H1975 NSCLC cells [4–7]. The protein HMGB1 is localized in the nucleus but when released into the extracellular space is highly immunogenic [6, 7]. Our prior work has shown that HMGB1 can be released into the extracellular environment by drug combinations that stimulate autophagosome formation. Combined knock down of HDACs 1+ 2, 1 + 3, 2 + 3 or 3 + 10 all modestly enhanced the expression of HMGB1.

**Figure 12:**
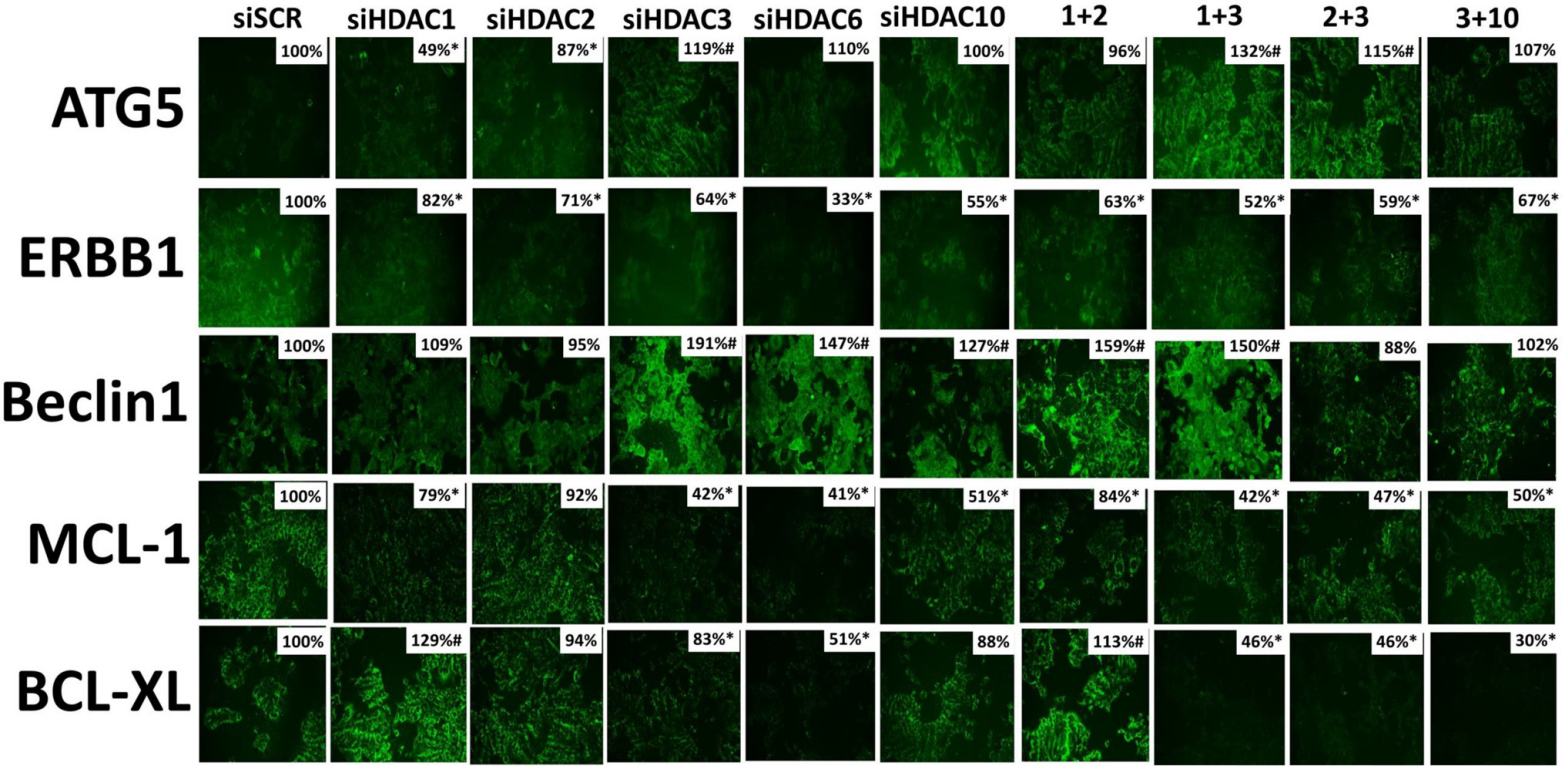
Knock down of HDAC expression reduces the levels of ERBB1, BCL-XL and MCL-1 expression in 4T1 cells 4T1 cells were transfected with an siRNA control (siSCR) or with siRNA molecules to knock down the expression of HDAC1, HDAC2, HDAC3, HDAC6, HDAC10, or with combinations of HDAC knock down as indicated in the Figure. Twenty-four h after transfection cells were fixed in place and immunostaining performed to determine the staining intensity under each knock down condition of ATG5, ERBB1, Beclin1, MCL-1 and BCL-XL. (data from multiple separate images & treatments ± SEM) ^*^p < 0.05 less than siSCR control; ^#^*p* < 0.05 greater than siSCR control.

In prior studies, we have demonstrated that [neratinib + valproate] reduces the expression of MCL-1 and BCL-XL, and enhances the expression of ATG5 and Beclin1, in an eIF2α -dependent fashion [1]. Thus, we next re-interrogated cells from our prior immunostaining studies to determine the impact knocking down HDAC expression had on the levels of ERBB1, Beclin1, ATG5, MCL-1 and BCL-XL. Inhibition of HDAC6 function modestly enhanced the expression of ATG5 and strongly enhanced the expression of Beclin1 whereas it reduced the expression of ERBB1, MCL-1 and BCL-XL (Figure 12). The expression of MCL-1 and BCL-XL were reduced by combined knock down of HDACs 1+2, 1+3 and 2+3. The expression of Beclin1 was strongly enhanced by knock down of HDACs 1+2 and 1+3. ERBB1 expression was significantly reduced by knock down of all HDACs, alone or in combination. Thus, in addition to having their expression levels modulated by drug-induced eIF2α signaling, the drug combination through modulation of HDAC expression and HDAC activity also acts to reduce the levels of ERBB1, MCL-1 and BCL-XL, and to enhance the levels of Beclin1 and ATG5.

## DISCUSSION

The present studies were performed to define the changes in the cell biology of 4T1 rodent TNBC tumors previously exposed to [neratinib + valproate]. Prior exposure of 4T1 tumors to the drug combination radically altered the protein expression profile of the surviving out-growth of 4T1 tumor cells. The surviving TNBC tumor cells grew more slowly than vehicle control treated tumors and had reduced their expression of ERBB1, N-RAS and K-RAS by over 50%. The re-grown cells expressed lower amounts of HDACs 1-3, 6 and 10. Loss of these HDACs was shown to be causal in reducing the expression of protective MCL-1 and BCL-XL and enhancing the expression of autophagosome-regulatory Beclin1 and ATG5. Furthermore, the altered expression profile of HDACs was also causal in the surviving tumor cells expressing less IDO-1, ODC and PD-L1 and expressing more Class I MHCA. These drug-induced changes in immunological biomarkers were associated with a greater intra-tumor infiltration of CD8+ cells and active T cells, active NK cells, and M1 macrophages. These observations correlated with [neratinib + valproate] enhancing the anti-tumor efficacy of an anti-PD1 checkpoint inhibitory antibody [1]. From these observations we conclude that [neratinib + valproate] treatment of mammary tumors may be an efficacious approach to both kill and immuno-sensitize the tumor to checkpoint inhibitory antibodies. In addition, based on our data examining the expression of MCL-1 and BCL-XL, we hypothesize that by adding a third agent, the BCL-2 inhibitor venetoclax, we may enhance the lethality of the [neratinib + valproate] drug combination. Studies beyond the scope of the present manuscript will be required to determine the usefulness of venetoclax in our system.

Previously we had demonstrated that neratinib as a single agent, and to a greater extent the [neratinib + valproate] combination, could rapidly reduce the expression of ERBB1-4 as well as of K-/N-RAS proteins [1, 2]. Using a series of PDX melanoma isolates, each expressing a similar level of mutant N-RAS, we wished to determine whether there was a simple correlation between the down-regulation of receptors and RAS proteins and the ability of [neratinib + valproate] to induce tumor cell killing. One isolate, that expressed less ERBB2 and ERBB3 than the other three isolates and that displayed the weakest down-regulation of mutant N-RAS by the drug combination also presented with the weakest cell death response to the drug combination. Another isolate that expressed twice as much ERBB2 and ERBB3 but also had a similar weaker down-regulation of mutant N-RAS was twice as sensitive to the drug combination. This suggests the levels of ERBB2 may be more predictive for the actions of [neratinib + valproate] than the down regulation of mutant N-RAS expression. However, in the other two isolates examined in our studies, that also expressed twice as much ERBB2 and ERBB3, but also exhibited twice as much mutant N-RAS down regulation, we observed high levels of drug combination lethality. These data would, conversely, argue that it is the amount of mutant N-RAS down regulation that would be predictive of [neratinib + valproate] lethality. Studies beyond the scope of the present manuscript will be required to understand the relative roles of ERBB2 and mutant RAS proteins in protecting tumor cells from [neratinib + valproate].

A three-day exposure of immune-competent mice to [neratinib + valproate] significantly suppressed 4T1 tumor growth, with drug treated tumors requiring an additional eight days to achieve the same tumor volume [1]. Drug treatment neither altered normal tissue cyto-architecture, animal body mass nor animal behavior. These findings argue that [neratinib + valproate] is likely to be safely tolerated in patients, and recently we have recently proposed to Puma Inc. and to VCU Massey Cancer Center that a new dose-escalation phase I trial in all solid tumors be performed combining neratinib and sodium valproate. Based on the biomarker data obtained in this manuscript and prior published studies we hope to be able to confirm that the [neratinib + valproate] combination can reduce RAS expression and modify the immunogenicity of tumors that would ultimately facilitate this drug combination being combined with anti-PD1 checkpoint inhibitory antibodies.

## MATERIALS AND METHODS

### Materials

Sodium valproate was from Sigma (St. Louis, MO). Neratinib was supplied by Puma Biotechnology Inc. (Los Angeles, CA). Trypsin-EDTA, DMEM, RPMI, penicillin-streptomycin were purchased from GIBCOBRL (GIBCOBRL Life Technologies, Grand Island, NY). SKOV3, OVCAR, HT29, HCT116, BT474, PANC1 cells were purchased from the ATCC and were not further validated beyond that claimed by ATCC. Cells were re-purchased every ∼6 months. Spiky ovarian cancer cells, an established PDX model, were kindly provided by Dr. Karen Paz (Champions Oncology, NJ). Mutant N-RAS expressing melanoma cells were provided by Dr. Kirkwood from the University of Pittsburgh cell isolate repository. Commercially available validated short hairpin RNA molecules to knock down RNA/protein levels were from Qiagen (Valencia, CA) (Supplementary Figure 2). Reagents/performance of experimental procedures were described in refs: 1, 2, 4–7.

### Methods

#### Culture and *in vitro* exposure of cells to drugs

All cell lines were cultured at 37°C (5% (v/v CO_2_) *in vitro* using RPMI supplemented with dialyzed 5% (v/v) fetal calf serum and 1% (v/v) Non-essential amino acids. For short term cell killing assays, immune-staining studies, cells were plated at a density of 3 × 10^3^ per cm^2^ and 24 h after plating treated with various drugs, as indicated. *In vitro* drug treatments were generally from a 100 mM stock solution of each drug and the maximal concentration of Vehicle carrier (VEH; DMSO) in media was 0.02% (v/v). Cells were not cultured in reduced serum media during any experiment in this manuscript. The safe achievable plasma C_max_ for neratinib is ∼150 nM and for sodium valproate ∼1 mM.

### Transfection of cells with siRNA or with plasmids

#### For plasmids

Cells were plated and 24 h after plating, transfected. Plasmids expressing a specific mRNA (or siRNA) or appropriate vector control plasmid DNA was diluted in 50 μl serum-free and antibiotic-free medium (1 portion for each sample). Concurrently, 2 μl Lipofectamine 2000 (Invitrogen), was diluted into 50 μl of serum-free and antibiotic-free medium (1 portion for each sample). Diluted DNA was added to the diluted Lipofectamine 2000 for each sample and incubated at room temperature for 30 min. This mixture was added to each well/dish of cells containing 200 μl serum-free and antibiotic-free medium for a total volume of 300 μl, and the cells were incubated for 4 h at 37°C. An equal volume of 2× medium was then added to each well. Cells were incubated for 24 h, then treated with drugs.

#### Transfection for siRNA

Cells from a fresh culture growing in log phase as described above, and 24 h after plating transfected. Prior to transfection, the medium was aspirated and serum-free medium was added to each plate. For transfection, 10 nM of the annealed siRNA, the positive sense control doubled stranded siRNA targeting GAPDH or the negative control (a “scrambled” sequence with no significant homology to any known gene sequences from mouse, rat or human cell lines) were used. Ten nM siRNA (scrambled or experimental) was diluted in serum-free media. Four μl Hiperfect (Qiagen) was added to this mixture and the solution was mixed by pipetting up and down several times. This solution was incubated at room temp for 10 min, then added drop-wise to each dish. The medium in each dish was swirled gently to mix, then incubated at 37°C for 2 h. Serum-containing medium was added to each plate, and cells were incubated at 37°C for 24 h before then treated with drugs (0–24 h). Additional immuno-fluorescence/live-dead analyses were performed at the indicated time points.

#### Colony formation/isobologram assays

Single cells were plated in 6-well plates in sextuplicate as individual cells (500 cells per well). After 12 h the cells were treated with vehicle control, neratinib, sodium valproate or the drugs combined, at the indicated concentrations in the figure, at a fixed ratio. After 24 h, the media is removed, the cells washed with warm drug-free media, and fresh drug-free media placed on the cells. After 10 days, colonies of > 50 cells have formed, and the cells are fixed in place and stained with crystal violet. The plating efficiency under each treatment condition is determined and the fraction affected determined (any group of cells > 50 cells is considered a colony). Synergy was determined using the Calcusyn for Windows program using the method of Cho and Talalay (*n* = 2 independent studies in sextuplicate). A combination index of less than 1.00 indicates a synergy of drug interaction. The Fraction affected data is plotted alongside the Combination Index.

### Detection of cell viability, protein expression and protein phosphorylation by immuno-fluorescence using a Hermes WiScan wide-field microscope

http://www.idea-bio.com/, Cells (4 × 10^3^) are plated into each well of a 96 well plate, and cells permitted to attach and grow for the next 18 h. Based on the experiment, after 18 h, cells are then either genetically manipulated, or are treated with drugs. For genetic manipulation, cells are transfected with plasmids or siRNA molecules and incubated for an additional 24 h. Cells are treated with vehicle control or with drugs at the indicated final concentrations, alone or in combination. Cells are then isolated for processing at various times following drug exposure. The 96 well plate is centrifuged/cyto-spun to associate dead cells (for live-dead assays) with the base of each well. For live dead assays, after centrifugation, the media is removed and cells treated with live-dead reagent (Thermo Fisher Scientific, Waltham MA) and after 10 min this is removed and the cells in each well are visualized in the Hermes instrument at 10× magnification. Green cells = viable; yellow/red cells = dying/dead. The numbers of viable and dead cells were counted manually from three images taken from each well combined with data from another two wells of separately treated cells (i.e. the data is the mean cell dead from 9 data points from three separate exposures).

For immuno-fluorescence studies, after centrifugation, the media is removed and cells are fixed in place and permeabilized using ice cold PBS containing 0.4% paraformaldehyde and 0.5% Triton X-100. After 30 min the cells are washed three times with ice cold PBS and cells are pre-blocked with rat serum for 3 h. Cells are then incubated with a primary antibody to detect the expression/phosphorylation of a protein (usually at 1:100 dilution from a commercial vendor) overnight at 37°C. Cells are washed three times with PBS followed by application of the secondary antibody containing an associated fluorescent red, green or blue chemical tag. After 3 h of incubation the antibody is removed and the cells washed again. A secondary alone negative control was included to assess background staining in the absence of primary antibody; at present, using mouse, rabbit and goat secondary antibodies the background staining effect in our system is essentially zero. The cells are visualized at either 10× or 60× in the Hermes microscope for imaging assessments of fluorescence intensity. All immunofluorescent images for each individual protein/phospho-protein are taken using the identical machine settings so that the levels of signal in each image can be directly compared to the level of signal in the cells treated with drugs. Imaging for fluorescence intensity is made using software integral to the Hermes microscope. Selections of groups of cells are made (∼40 in each well) from each treatment in triplicate and the mean intensity determined. The software automatically assesses and removes background fluorescence from each data set so that comparisons are made examining actual cell staining intensity between conditions/treatments. For images of 5 μm tumor sections, the staining intensity data are the mean of multiple random fields from multiple slides (± SEM).

For presentation, the enhancement of image brightness/contrast is processed at 9999 dpi using Adobe Photoshop CS6 and is simultaneously performed for each individual set of protein/phospho-protein to permit direct comparison of the image intensity between treatments. Images are labeled and figures generated in Microsoft PowerPoint.

### Detection of cell death by trypan blue assay

Cells were harvested by trypsinization with Trypsin/EDTA for ∼10 min at 37°C. Harvested cells were combined with the culture media containing unattached cells and the mixture centrifuged (800 rpm, 5 min). Cell pellets were resuspended in PBS and mixed with trypan blue agent. Viability was determined microscopically using a hemocytometer. Five hundred cells from randomly chosen fields were counted and the number of dead cells was counted and expressed as a percentage of the total number of cells counted.

### Assessment of autophagy

Cells were transfected with a plasmid to express a green fluorescent protein (GFP) and red fluorescent protein (RFP) tagged form of LC3 (ATG8). For analysis of cells transfected with the GFP-RFP-LC3 construct, the GFP/RFP-positive vesicularized cells were examined under the ×40 objective of a Zeiss Axiovert fluorescent microscope.

### Data analysis

Comparison of the effects of various treatments (performed in triplicate three times) was using one-way analysis of variance and a two tailed Student’s *t*-test. Statistical examination of *in vivo* animal survival data utilized both a two tailed Student’s *t*-test and log rank statistical analyses between the different treatment groups. Differences with a *p*-value of < 0.05 were considered statistically significant. Experiments shown are the means of multiple individual points from multiple experiments (± SEM).

## SUPPLEMENTARY MATERIALS FIGURES



## Abbreviations

ERK: extracellular regulated kinase
PI3K: phosphatidyl inositol 3 kinase
ca: constitutively active
dn: dominant negative
ER: endoplasmic reticulum
mTOR: mammalian target of rapamycin
MAPK: mitogen activated protein kinase
PTEN: phosphatase and tensin homologue on chromosome ten
ROS: reactive oxygen species
CMV: empty vector plasmid or virus
si: small interfering
SCR: scrambled
VEH: vehicle
HDAC: histone deacetylase
