# Supplementary material for: [Neratinib + Valproate] exposure permanently reduces ERBB1 and RAS expression in 4T1 mammary tumors and enhances M1 macrophage infiltration
Source: Oncotarget. 2017 Dec 26;9(5):6062–74. doi: 10.18632/oncotarget.23681 (PMC5814195; doi:10.18632/oncotarget.23681)
Supplement: Supplementary file 1 [file oncotarget-09-6062-s001.pdf]

[Neratinib + Valproate] exposure permanently reduces ERBB1 and RAS expression in 4T1 mammary tumors and enhances M1 macrophage infiltration

SUPPLEMENTARY MATERIALS

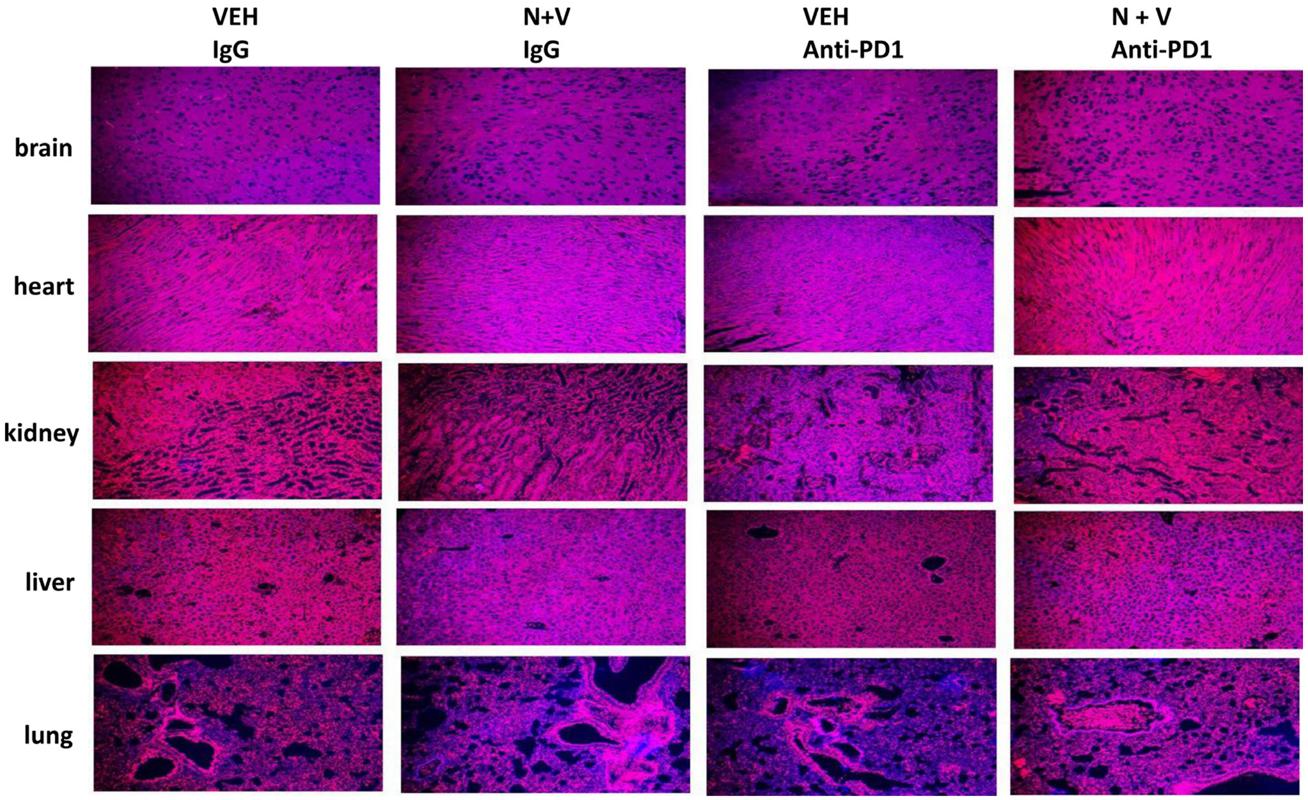

Supplementary Figure 1: Prior [neratinib + valproate] exposure does not cause frank obvious normal tissue damage in BALB/c mice at Day 16. A. BALB/c mice carrying 4T1 tumors were humanely sacrificed on Day 16. Organs were harvested from the mice and fixed, paraffin embedded and sectioned (5 µm). Sections were renatured, blocked and H&E performed to determine the tissue morphology of each of the normal tissues examined. Images were taken at 10× magnification.

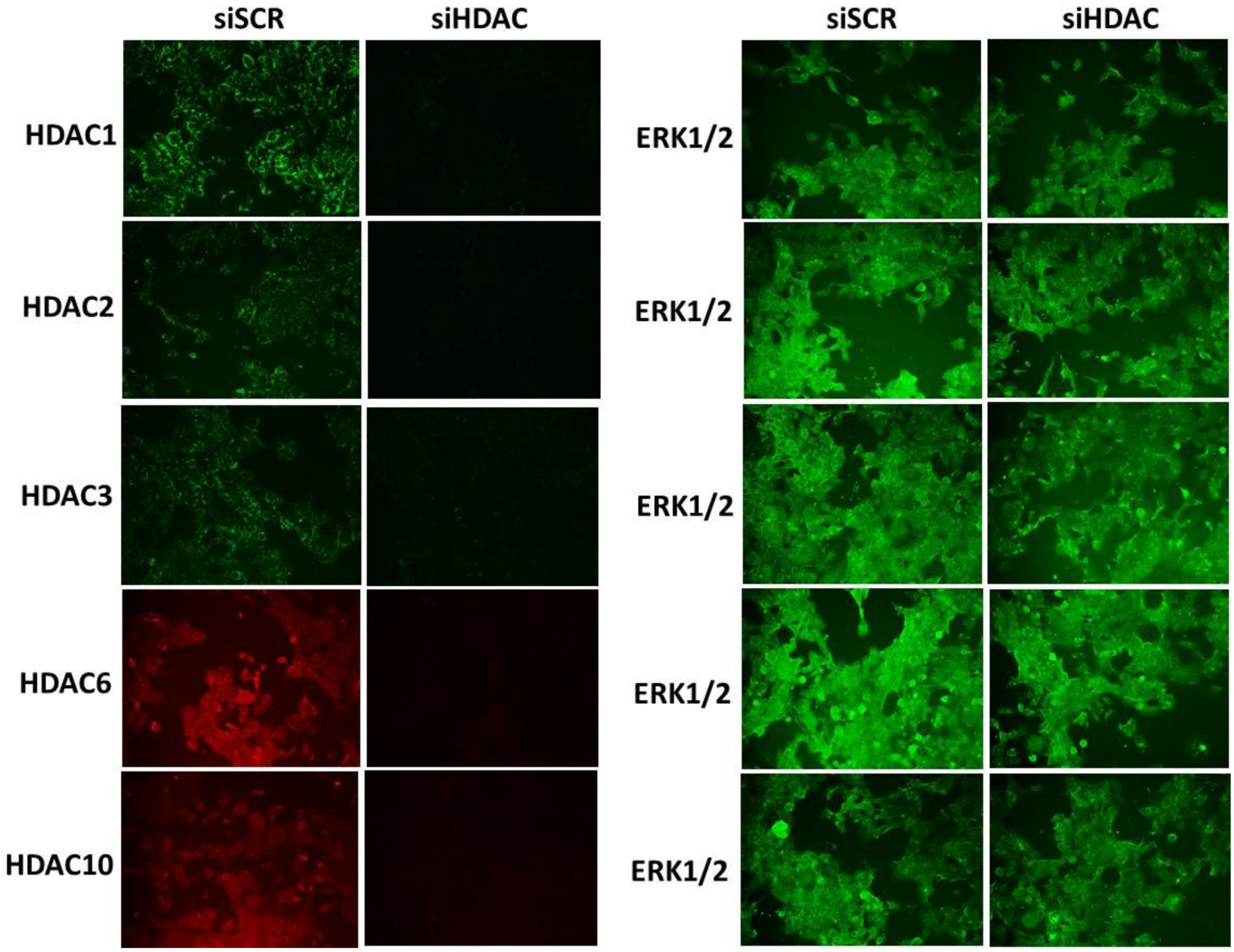

Supplementary Figure 2: Control IF images showing knock down of HDAC proteins in 4T1 cells.
